# Isolated First Septal Perforator Spontaneous Coronary Artery Dissection Causing Acute Myocardial Infarction

**DOI:** 10.1016/j.jaccas.2026.107931

**Published:** 2026-04-16

**Authors:** Ryan A. Karlsson, John P. Birrane, Rory O'Hanlon, Andrew O. Maree

**Affiliations:** a Department of Cardiology, St James's Hospital, Dublin, Ireland; b Cardiovascular MRI Unit, Blackrock Clinic, Dublin, Ireland

**Keywords:** cardiac magnetic resonance, first septal perforator, literature review, spontaneous coronary artery dissection

## Abstract

**Background:**

Isolated spontaneous coronary artery dissection (SCAD) of the first septal perforator (S1) is rare.

**Case Summary:**

A 48-year-old man presented with chest pain after physical exertion. Coronary angiography demonstrated type 1 SCAD confined to S1. Cardiac magnetic resonance (CMR) confirmed a transmural septal infarction. The patient was managed conservatively with antiplatelet and beta-blocker therapy and experienced an uncomplicated recovery.

**Discussion:**

A limited number of cases of isolated S1 SCAD have been previously reported, predominantly affecting women and all managed conservatively. To our knowledge, this case represents the youngest reported male patient and appears to be the first reported case demonstrating CMR-confirmed transmural septal infarction secondary to type 1 S1 SCAD. Optimal long-term medical therapy remains undefined.

**Take-Home Messages:**

SCAD should be considered in patients presenting with chest pain after physical or emotional stress. CMR is a valuable adjunct after angiography in cases of diagnostic uncertainty.

## Visual Summary

**Figure:**
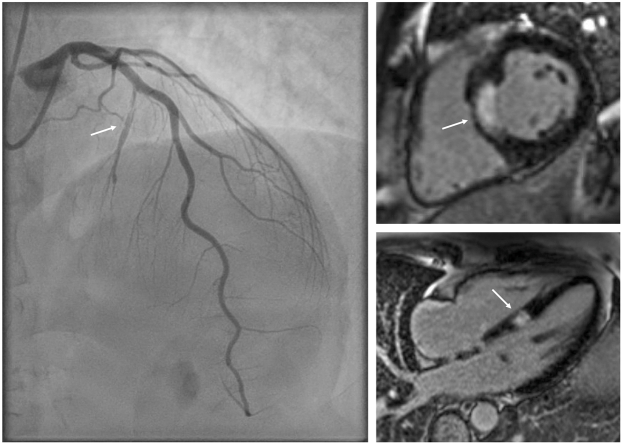

## History of Presentation

A previously well 48-year-old man presented with 2 days of chest pain radiating to the left arm, associated with dyspnea and vomiting. He reported heavy lifting several hours before the onset of symptoms. Clinical examination revealed hypertension (141/89 mm Hg), tachycardia (103 beats/min) and low-grade pyrexia. Heart sounds were dual with no audible murmur, and the chest was clear to auscultation. The calves were nontender bilaterally, and he was clinically euvolemic.

**Take-Home Messages**

• Spontaneous coronary artery dissection should be considered in patients who present with acute chest pain after physical or emotional stress.
• Given the rarity of acute myocardial infarction secondary to isolated first septal perforator spontaneous coronary artery dissection, cardiac magnetic resonance represents a useful confirmatory test.

## Past Medical History

His background was significant for dyslipidemia and prior spontaneous left-sided pneumothorax, managed 13 years previously with video-assisted thoracoscopic surgery. There was no family history of connective tissue disorder, arteriopathy, or hypermobility syndrome. He was an active smoker with a 10-pack-year history and consumed no alcohol.

## Differential Diagnosis

Differential diagnoses for the patient's presentation include acute myocardial infarction, acute pericarditis or myocarditis, pneumothorax, pulmonary embolus, and respiratory tract infection.

## Investigations

**Figure 1:**
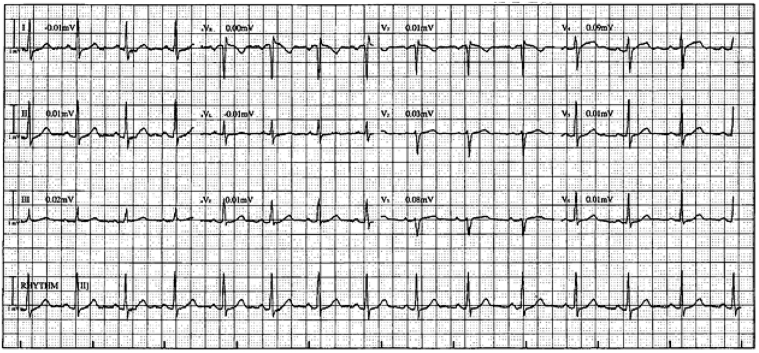
12-Lead Electrocardiogram 12-lead electrocardiogram showing sinus rhythm, abnormal precordial R-wave progression, mild inferolateral ST-segment depression, and mild ST-segment elevation <1 mm in leads aVR, V_1_, and V_3_.

The 12-lead electrocardiogram showed sinus rhythm, abnormal precordial R-wave progression, mild inferolateral ST-segment depression, and mild ST-segment elevation <1 mm in leads aVR, V_1_, and V_3_ (Figure 1).

Blood tests revealed a serum hemoglobin of 16.6 g/dL (reference range: 13.0-17.0 g/dL), C-reactive protein level of 60 mg/L (reference range: <10 mg/L), and erythrocyte sedimentation rate of 21 mm/h (reference range: 0-10 mm/h). Serum high-sensitivity troponin I returned at 11,626 ng/L and later peaked at 12,774 ng/L (reference range: <53 ng/L). Serum N-terminal pro–B-type natriuretic peptide was 770 ng/L (reference range: <400 ng/L). Renal profile and liver enzymes were normal. Chest radiograph revealed normal cardiac size and expected post–video-assisted thoracoscopic surgery changes at the left lung apex. Transthoracic echocardiography demonstrated a structurally normal heart with preserved left ventricular (LV) function and no regional wall motion abnormalities.

## Management

**Figure 2:**
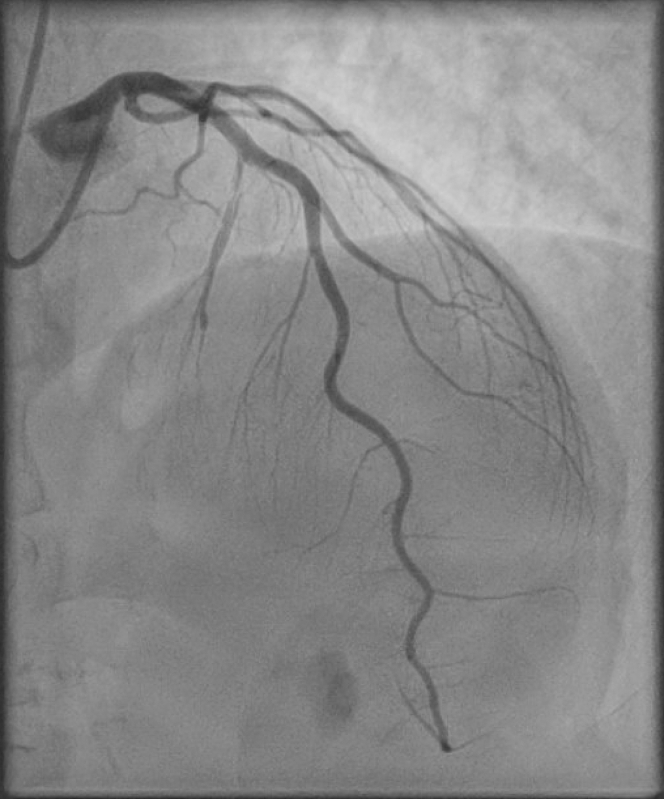
Coronary Angiography Coronary angiography (postero-anterior cranial view) of the left anterior descending artery demonstrating type 1 spontaneous coronary artery dissection of the first septal perforator branch, with contrast staining of the arterial wall, dye hang-up, and multiple radiolucent lumina.

**Figure 3:**
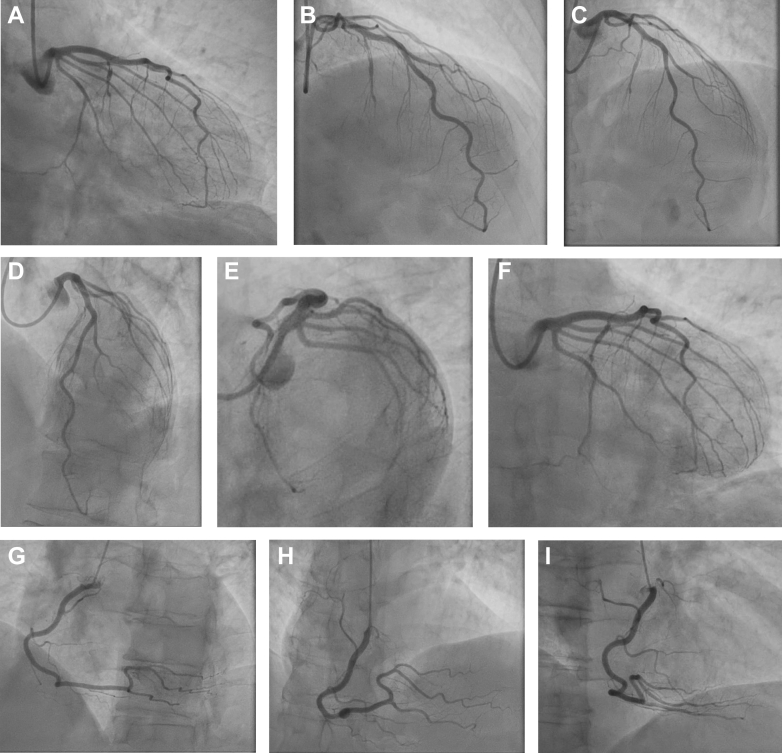
Coronary Angiography Complete Series of Views Left coronary artery (A) RAO caudal, (B) RAO cranial, (C) PA cranial, (D) LAO cranial, (E) LAO caudal, (F) PA caudal views, and right coronary artery (G) LAO, (H) PA cranial, and (I) RAO views. There is a dominant right coronary artery with no obstructive disease. Type 1 spontaneous coronary artery dissection of the first septal perforator artery is visible. There is notable absence of disease affecting the remainder of the left coronary system. LAO = left anterior oblique; PA = postero-anterior; RAO = right anterior oblique.

In the setting of an acute coronary syndrome (ACS) presentation, dual antiplatelet therapy (DAPT) with aspirin and ticagrelor was administered along with high-dose atorvastatin therapy. The patient was transferred to the tertiary cardiology center for urgent coronary artery assessment. Coronary angiography revealed a large-caliber first septal perforator with evidence of proximal coronary artery dissection and contrast hang-up (Figure 2, Video 1). There was otherwise normal anatomy with the remaining coronary arteries free from disease (Figure 3). Percutaneous coronary intervention (PCI) was not carried out.

**Figure 4:**
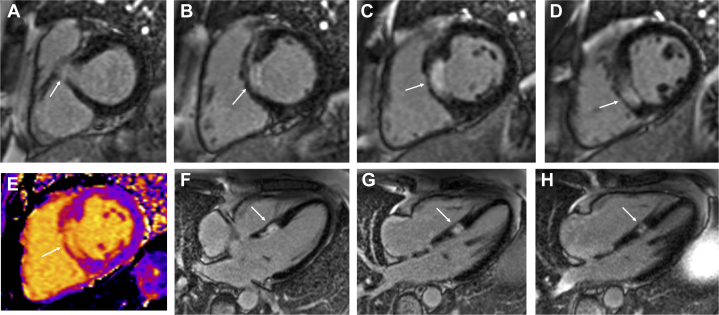
Cardiac Magnetic Resonance Imaging Cardiac magnetic resonance short-axis late gadolinium enhancement images (A to D), short-axis T1 map (E), and 4-chamber late gadolinium enhancement images (F to H) demonstrating transmural infarction of the septum at basal and mid-ventricular levels. There is no evidence of microvascular obstruction, myocardial edema, or pericardial effusion. Arrows indicate the infarct location.

Cardiac magnetic resonance (CMR) was performed to confirm the diagnosis. This revealed isolated akinesis of the septum at basal and mid-ventricular levels with corresponding late gadolinium enhancement consistent with a transmural infarction (Figure 4, Video 2). There were normal indexed LV volumes and overall mild LV systolic impairment, and no evidence of microvascular obstruction, myocardial edema, or pericardial effusion.

Follow-up computed tomography angiography from the aortic arch to the vertex and assessing the renal vasculature showed no arterial abnormality or evidence of fibromuscular dysplasia.

## Outcome and Follow-Up

The patient was discharged home 4 days after coronary angiography. DAPT was prescribed for 3 months to mitigate the risk of superimposed luminal thrombosis at the site of coronary dissection during the acute phase. In the absence of stenting, aspirin monotherapy was planned thereafter, reflecting a commonly adopted approach in conservatively managed spontaneous coronary artery dissection (SCAD). Low-dose bisoprolol therapy was added. In the context of prior of spontaneous pneumothorax, the patient was referred for rheumatology assessment regarding the potential for an underlying connective tissue disorder. At 12-week follow-up, he reported no recurrence of symptoms. Follow-up surveillance CMR is planned to reassess for the interval development of septal thinning or ventricular septal defect.

## Discussion

SCAD is defined as a noniatrogenic, nontraumatic separation of the coronary arterial walls that creates a false lumen between the intima and media or between the media and adventitia, which may compress the true coronary lumen and cause myocardial ischemia or infarction.^1^ Study consensus has suggested 2 likely mechanisms of SCAD: the predominant being primary intramural hematoma formation (possibly secondary to primary rupture of the vasa vasorum) and the alternative recognized mechanism being that of a primary tear of the arterial intima.^2^ Recognized risk factors for the development of SCAD include female gender, pregnancy, fibromuscular dysplasia, connective tissue disorder, cocaine use, and familial predisposition.^1^ Intense emotional, physical, or Valsalva-type stressors are also thought to play a role in susceptible individuals.^3^ Despite advances in intravascular imaging techniques and increased awareness among physicians, SCAD most likely remains an under-recognized cause of acute myocardial infarction.^4^ Its reported prevalence in ACS is currently estimated at 2.1%.^5^ Although SCAD typically affects the epicardial coronary arteries, isolated involvement of intramyocardial vessels such as the first septal perforator is exceptionally rare and may be easily overlooked on coronary angiography.^4^^,^^6^

The Saw angiographic classification remains the most widely adopted system for categorizing SCAD^7^ and describes 4 subtypes (types 1-4), each with characteristic angiographic features, with type 2 SCAD being the most common. Intracoronary imaging should be used with caution in cases of diagnostic uncertainty given the risk of dissection propagation with instrumentation.^2^^,^^4^ Coronary computed tomography angiography offers a noninvasive option for coronary assessment however generally lacks the resolution to exclude SCAD; therefore, its current value lies predominantly in follow-up imaging to demonstrate vessel healing.^2^ PCI for SCAD has been associated with lower technical success and suboptimal outcomes when compared with PCI for atherosclerotic disease.^8^^,^^9^ The risk of PCI-related complications is higher including inadvertent dilatation and/or stenting within the false lumen, dissection propagation, stent underexpansion due to subsequent intramural hematoma resorption, and iatrogenic coronary dissection or perforation. PCI should therefore be reserved for high-risk cases that include proximal coronary occlusion, persistent ischemia, or hemodynamic or electrical instability.^5^

Medical therapy for SCAD follows the recommendations for management of ACS; however, varying levels of evidence exist to support the use of conventional therapies. Beta-blockers have been associated with a reduction in the incidence of recurrent SCAD^10^; however, the optimal duration for their use remains undefined. Similarly, the efficacy and optimal duration of single-antiplatelet therapy vs DAPT for conservatively managed SCAD has not been established.^2^ Furthermore, no study to date has demonstrated a positive or harmful effect of statin use after SCAD, suggesting that its role may be only in the management of patients found to have dyslipidemia. The overall lifetime risk of SCAD recurrence has been reported at up to 30%,^1^ and may be predicted by an underlying history of hypertension.^10^

A total of 15 prior cases of SCAD involving the first septal perforator alone have been described in the literature. Supplemental Table 1 summarizes the key features of each reported case. A total of 80% of prior cases affected females, and 53% were associated with a preceding physical or emotional stressor. All cases were diagnosed using invasive coronary angiography and managed conservatively, with intravascular imaging also used in 2 cases. Recurrent myocardial infarction within 3 months was reported at a rate of 13%. Routine follow-up imaging strategies were heterogenous across cases. Screening for fibromuscular dysplasia was performed in 9 of the reported cases with the condition identified in 67% of those screened. To our knowledge, this case represents the youngest reported male patient with SCAD confined to the first septal perforator and appears to be the first reported case demonstrating CMR-confirmed transmural septal infarction secondary to type 1 SCAD isolated to this vessel.

Transmural infarction involving the interventricular septum may have important clinical implications given the septum's structural role and the proximity of the cardiac conduction system. Infarction involving the first septal perforator territory may predispose to atrioventricular conduction disturbance, bundle branch block, ventricular arrhythmia, septal dysfunction, myocardial thinning, and, in severe cases, ventricular septal rupture.^11^ Identification of septal involvement at coronary angiography and on subsequent noninvasive imaging is therefore clinically important.

**Visual Summary:**
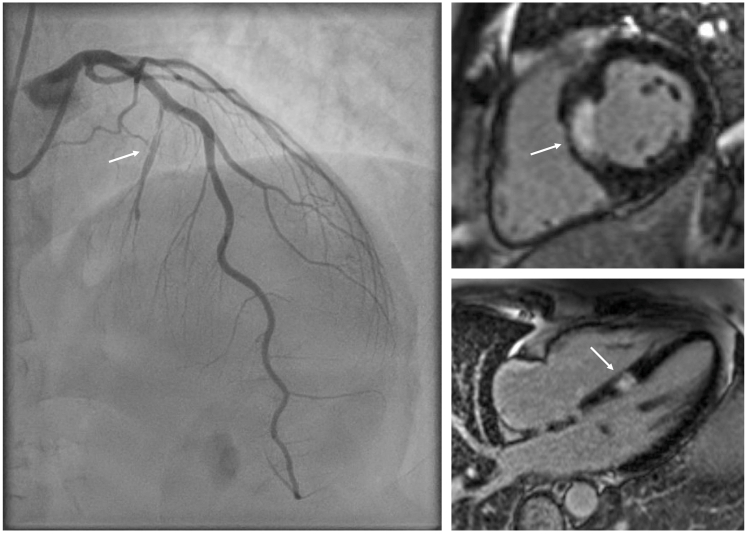
Isolated First Septal Perforator Spontaneous Coronary Artery Dissection Causing Acute Myocardial Infarction Type 1 spontaneous coronary artery dissection of the first septal perforator resulting in transmural infarction of the interventricular septum (arrows indicate the culprit vessel on angiography and corresponding infarct on cardiac magnetic resonance imaging).

## Conclusions

This case serves as a reminder to consider the first septal perforator as an isolated culprit vessel in ACS secondary to SCAD. Although conservative management represents the recommended approach, further studies are warranted to define specific optimal medical therapy and follow-up imaging strategy for this condition.

## Funding Support and Author Disclosures

The authors have reported that they have no relationships relevant to the contents of this paper to disclose.

## Appendix

Video 1 Coronary Angiography (right anterior oblique Caudal) Cine Imaging of the Left Coronary Artery

Video 2 Cardiac Magnetic Resonance Short-Axis Cine Imaging

Supplemental Table 1 Summary of all prior reported cases of isolated first septal perforator spontaneous coronary artery dissection
