# Supplementary material for: Isolated First Septal Perforator Spontaneous Coronary Artery Dissection Causing Acute Myocardial Infarction
Source: JACC Case Rep. 2026 Apr 16;31(21):107931. doi: 10.1016/j.jaccas.2026.107931 (PMC13221823; doi:10.1016/j.jaccas.2026.107931)
Supplement: Supplemental Table 1 — Summary of all prior reported cases of isolated first septal perforator spontaneous coronary artery dissection [file mmc3.docx]

**Supplemental Table 1. Summary of all prior reported cases of isolated first septal perforator spontaneous coronary artery dissection**

| Authors (Year) | Clinical Presentation | Peak Troponin  (reference range) | Diagnostic Modality | SCAD Angiographic Classification | Treatment Strategy  (specific medical therapy) | Clinical Outcome | Routine follow-up imaging |
| --- | --- | --- | --- | --- | --- | --- | --- |
| Tanimura et al. ^1^  (2025) | 61F with NSTEMI  Hypertensive  Physical stressor (defecation) | 0.012ng/mL (<0.014ng/mL) | ECG-gated contrast-enhanced CT  Invasive coronary angiography  IVUS | Type 2 | Conservative  (aspirin, nifedipine, nicorandil, statin) | Discharged day 4  No recurrence at one month | CCTA |
| Rezkalla et al. ^2^  (2023) | 68F with NSTEMI  Hypertensive  Emotional stressor (husband’s illness) | 1,308ng/L  (<11ng/L) | Invasive coronary angiography  CMR | Type 2 | Conservative  *(not specified)* | Discharged day 2  Recurrent MI on day 4  No subsequent recurrence at five months | Imaging assessment for FMD (negative) |
| Sharkey et al. ^3^  (2021) | Series of 11 cases  38–64 years;  82% female;  100% NSTEMI  Physical or emotional stressor in 55%  54% hypertensive on presentation | Median 4.02  [IQR 1.49–12.38], range 0.68–406ng/mL  (<0.056ng/mL) | Invasive coronary angiography  CMR (45%) | Type 1 (27%)  Type 2 (64%)  Type 3 (9%) | Conservative (100%)  *(not specified)* | Median length-of-stay: 4 days  [IQR 2-6]  Recurrent MI in 2 (18%); occurring at two months and seven years respectively | CT angiography assessment for FMD performed in 64%  (positive finding in 71% of scans) |
| Bastante et al. ^4^  (2019) | 63F with NSTEMI | *Not reported* | Invasive coronary angiography | Type 3 | Conservative  *(not specified)* | Discharged day three  No recurrence at 3 months | ICA  Imaging assessment for FMD (positive) |
| Chasseriaud et al. ^5^  (2016) | 53M with NSTEMI | 13.5ng/mL (<0.04ng/mL) | Invasive coronary angiography  OCT  CMR | Type 2 | Conservative  (aspirin, beta blocker, statin) | Discharged day two  No recurrence at 3 months | CMR at 3 months |

CCTA, coronary computed tomography angiography; CMR, cardiac magnetic resonance; ECG, electrocardiogram; F, Female; FMD, fibromuscular dysplasia; ICA, invasive coronary angiography; IQR, interquartile range; IVUS, intravascular ultrasound; M, Male; NSTEMI, non-ST-elevation myocardial infarction; OCT, optical coherence tomography; SCAD, spontaneous coronary artery dissection

**References**

1. Tanimura K, Matsutera R, Nakajima K, Takaoka H. Acute myocardial infarction due to isolated spontaneous coronary artery dissection in the first septal branch: a case report. European Heart Journal - Case Reports 2025;9.

2. Rezkalla JA, Lathiya MK, Araoz PA, Singh G, Tweet MS. Acute Myocardial Infarction Caused by Spontaneous Coronary Artery Dissection of the First Septal Perforator. JACC: Case Reports 2023;14:101833.

3. Sharkey SW, Alfadhel M, Thaler C et al. Recognition of acute myocardial infarction caused by spontaneous coronary artery dissection of first septal perforator. European Heart Journal Acute Cardiovascular Care 2021;10:933-939.

4. Bastante T, García-Guimaraes M, Rivero F, Antuña P, Cuesta J, Alfonso F. Isolated septal branch lesion as the only diagnostic clue for spontaneous coronary artery dissection. Coronary Artery Disease 2020;31.

5. Chasseriaud W, Tearney GJ, Montaudon M, Fialon B, Coste P, Gerbaud E. Isolated septal myocardial infarction due to spontaneous coronary artery dissection. Int J Cardiol 2016;212:259-61.
